# Increased throughput and ultra-high mass resolution in DESI FT-ICR MS imaging through new-generation external data acquisition system and advanced data processing approaches

**DOI:** 10.1038/s41598-018-36957-1

**Published:** 2019-01-09

**Authors:** Pieter C. Kooijman, Konstantin O. Nagornov, Anton N. Kozhinov, David P. A. Kilgour, Yury O. Tsybin, Ron M. A. Heeren, Shane R. Ellis

**Affiliations:** 1 Maastricht University, Maastricht Multimodal Molecular Imaging Institute (M4I), Maastricht, 6229ER The Netherlands; 2 TI-COAST, Amsterdam, 1098 XH The Netherlands; 3 Spectroswiss, EPFL Innovation Park, 1015 Lausanne, Switzerland; 4 Nottingham Trent University, Department of Chemistry and Forensics, Nottingham, NG11 8NS United Kingdom

## Abstract

Desorption electrospray ionisation-mass spectrometry imaging (DESI-MSI) is a powerful imaging technique for the analysis of complex surfaces. However, the often highly complex nature of biological samples is particularly challenging for MSI approaches, as options to appropriately address molecular complexity are limited. Fourier transform ion cyclotron resonance mass spectrometry (FT-ICR MS) offers superior mass accuracy and mass resolving power, but its moderate throughput inhibits broader application. Here we demonstrate the dramatic gains in mass resolution and/or throughput of DESI-MSI on an FT-ICR MS by developing and implementing a sophisticated data acquisition and data processing pipeline. The presented pipeline integrates, for the first time, parallel ion accumulation and detection, post-processing absorption mode Fourier transform and pixel-by-pixel internal re-calibration. To achieve that, first, we developed and coupled an external high-performance data acquisition system to an FT-ICR MS instrument to record the time-domain signals (transients) in parallel with the instrument’s built-in electronics. The recorded transients were then processed by the in-house developed computationally-efficient data processing and data analysis software. Importantly, the described pipeline is shown to be applicable even to extremely large, up to 1 TB, imaging datasets. Overall, this approach provides improved analytical figures of merits such as: (i) enhanced mass resolution at no cost in experimental time; and (ii) up to 4-fold higher throughput while maintaining a constant mass resolution. Using this approach, we not only demonstrate the record 1 million mass resolution for lipid imaging from brain tissue, but explicitly show such mass resolution is required to resolve the complexity of the lipidome.

## Introduction

The field of direct surface analysis mass spectrometry has seen a tremendous growth in the last decade, particularly in methods that enable analysis to be performed in the open environment at atmospheric pressure^1–3^. These methods require minimal-to-no sample preparation but can still provide detailed chemical information in short time. Applications in forensics, security, food industry and medical diagnostics have already been demonstrated^4–9^.

However, the amount of spectral information provided by these methods can also pose a challenge, particularly in the analysis of complex biological samples. The sheer number of chemical components can quickly overwhelm the resolving power of the mass spectrometer. This becomes a major consideration when ambient mass spectrometry is used in imaging approaches, referred to as mass spectrometry imaging (MSI), for instance to study the spatial distributions of isobaric molecules in complex samples such as biological tissues that can have distinct biochemical functionalities^10^. In MSI no sample fractionation (i.e. LC-MS) is possible so the resulting spectra are often complex and contain many isobaric ion signals. Selective approaches, such as derivatization or multiple reaction monitoring, are undesirable for discovery studies which are currently the main strength of MSI. Furthermore, spectral simplification, by reducing the number of ion adduct species (e.g., protonated or sodiated) through washing steps^11,12^, can add to sample preparation time and introduces the risk of analyte delocalisation.

For this reason, the coupling of high mass resolving power analysers — namely ion cyclotron resonance (ICR) and Orbitrap Fourier transform (FT) instruments — to imaging interfaces has received particular attention. For example, Dilillo *et al*. have recently applied FT-ICR MSI with matrix-assisted laser desorption/ionisation (MALDI) to study both intact proteins and metabolites in glioblastomas. This approach enabled them to separate both isobaric protein and metabolite signals^13^, while Cornett *et al*. demonstrated the power of MALDI FT-ICR MSI to unambiguously image drugs in tissues^14^. Various studies have also coupled ambient imaging methods such as desorption electrospray ionisation (DESI)^15–17^ — the most widely used ambient MSI technique^18^ — and laser ablation electrospray ionisation (LAESI)^19–21^, with Orbitrap and ICR-based FTMS instrumentation. FTMS resolution and mass accuracy can enable assignment of elemental or sum-composition formula to detected ions (including those with isotopic tags), thus providing detailed information regarding the local chemical composition of the sample.

Routine ICR and Orbitrap FTMS instrumental methods can, nevertheless, often be insufficient to distinguish certain isobaric ion pairings. A powerful example of this occurs in lipidomics where, for example, one requires a mass resolution of >150,000 to fully resolve lipid signals containing 2 atoms of ^13^C from the monoisotopic ion of analogous lipids with one fewer site of unsaturation (*Δm* = 8.9 mDa)^22^. Even higher mass resolution is required to resolve sodiated lipid ions from the corresponding protonated lipid containing two more carbons and three more double bonds in the acyl chains (e.g., [PC(34:1) + Na]^+^ vs [PC(36:4) + H]^+^, *Δm* = 2.4 mDa). Shevchenko *et al*. have recently shown that ultra-high resolution performance capable of baseline-resolving peaks of ^13^C isotopes of unlabelled and monoisotopic peaks of ^15^N labelled lipids (*Δm* = 6.3 mDa) opens new avenues for quantitative shotgun lipidomics^23^. FTMS based instruments are able to tackle these challenges by simply increasing the time-domain signal (transient) recording time, which has an (approximately) linear correlation to mass resolution. For example, extending the ion detection time from one to five seconds will result in a theoretical five-fold improvement in mass resolution. However, given that a typical MS image can consist of tens of thousands of pixels, acquisition time may become a constraining factor. Therefore, mass resolution is often sacrificed in favour of throughput^24^.

In this paper we present an approach to significantly improve the experimental mass resolution and accuracy per time unit in FT-ICR based MS imaging, enabling MS imaging with unparalleled mass resolving power. To achieve this result, we developed a novel architecture of a data acquisition system for time-domain signals (transients) recording from FTMS instruments and an advanced data processing pipeline to maximize the extraction of accurate mass spectral information from these transients. The developed data acquisition and processing pipeline targets the following procedures and capabilities: (i) parallel ion accumulation and detection for transients of any length; (ii) absorption mode FT post-processing adapted for big data processing; (iii) parallel (multi-core) processing of an image dataset without its splitting into a number of smaller chunks or using a “binning” method, and (iv) efficient internal re-calibration. The current state-of-the-art in these four domains is briefly summarized below.

## Parallel ion accumulation and detection

Parallel ion accumulation and detection refers to an ability of FTMS instruments to record a transient in an FTMS cell for a given scan during ion accumulation in another ion trap for the following scan. In cases where ion accumulation and ion detection times are comparable, for example both of them are about 0.1–1 s, this capability can significantly increase the throughput of FTMS measurements. Historically, perhaps the first implementation of parallel ion detection and accumulation was realized when two ICR cells were placed in a high magnetic field and operated independently^25^. The dual cell configuration allowed to accumulate ions in one cell while ion detection was taking place in the second cell. More recently, Bruce and co-workers developed this original idea into a more sophisticated configuration that included an array of ICR cells^26^. Up to date, none of these configurations has been realized in a commercial instrument and has not been employed for MSI experiments. The second type of parallel ion accumulation and detection capability is based on the use of ion accumulation in ion traps external to the magnetic field with a parallel ion detection taking place in the ICR cell for a pre-defined ion detection time. The up-to-date implementation of this approach relied on the data acquisition systems that allowed recording of transients for a certain pre-defined duration, e.g., 128 ms, 256 ms, 512 ms, *etc*. The increment of the length of these transients followed the 2-fold rule which follows from the architecture of the data acquisition systems. Therefore, in cases where the ion accumulation time exceeds a pre-defined transient length for a certain scan, there will be no ion detection taking place during the excess time. The first report of an experimental implementation of this approach to maximize the measurement duty cycle appeared around the same time as the dual-trap concept as a result of developments by Senko and co-workers^27^. A decade later, the same laboratory reported on the implementation of a higher performance data acquisition system that facilitated the overall experiment control and expanded the range of capabilities^28^. Interestingly, being implemented in FT-ICR MS for the past 20 years according to the published reports^27,29^, it is not explicitly mentioned as an employed method in the peer-reviewed papers. Furthermore, the only use of this capability in a commercial FT-ICR MS instrument, where it is known to be realized as an “accumulation during detect”, or ADD function (FT-ICR MS from Bruker Daltonics), has been mentioned in a conjunction with a related technology of ion mobility^30^. It should be noted, that the modern generations of Orbitrap FTMS instruments are capable of routinely performing parallel ion accumulation and detection in the way described above. Implementation of parallel ion accumulation and detection in the present work principally differs from the prior art by using the high-performance data acquisition system. The “high-performance” terminology is usually employed to signify the use of the new-generation electronics components with embedded advanced capabilities for in-line digital signal processing^31^. As shown below, the high-performance data acquisition systems allow transient detection for any duration and for all the time ions are trapped inside of the ICR cell. That means a fully parallel ion detection to external ion accumulation, fragmentation and eventual overheads. With respect to a DESI MSI application it means that any ion accumulation period can be matched with the ion detection period, without a need to adjust ion accumulation to a pre-defined transient length.

In case ultra-high resolution is required from an FTMS instrument, transient length can be increased up to 2–10 seconds and more, thus significantly shifting the scan rate principal contribution from ion accumulation to ion detection. Long transients and thus low scan rates are particularly detrimental to MSI due to a large number of pixels to constitute an image. Therefore, further measures to reduce the transient length without the loss of a resolution can be considered. In modern FTMS these methods include increasing the magnetic fields strength, up to 21T presently^32^, performing frequency multiples measurements, with the experimentally implemented quadruple frequency multiple FT-ICR MS operation as the highest one^33,34^, and absorption mode FT ICR MS (see below).

## Absorption mode Fourier transform MSI

Efficient processing of transients in modern FTMS implies spectral representation in absorption mode FT (aFT), which can be compared with the magnitude mode FT (mFT) spectra representation^35–38^. This switch provides a theoretical improvement in mass resolution of up to 2-fold, and an improvement in both signal-to-noise ratio and the mass accuracy for the same transient length^39^. So far, Smith *et al*. have provided the only report on the use of absorption mode FT-ICR for MSI. The data presented a mass resolving power of ~300,000 at *m/z* 700 in a MALDI approach with a scan time per pixel of 1.8 s using a 9.4T magnet, which is one of the highest resolutions reported for any MSI approach^40^. Some spectral features were still unresolved, which confirms the need for further improvements in MSI mass resolution. Absorption mode processing has not been regularly applied to MSI datasets because the procedure can be rather complex and time consuming. With newly developed data processing software we demonstrate the first implementation of absorption mode FT for FT-ICR MS imaging in combination with ambient ionization technologies (DESI), including processing of very large, up to 1 TB, datasets.

## Pixel-based internal recalibration

Conversion of frequency spectra represented in the aFT mode into accurately calibrated mass spectra is another challenge in FT-based MSI. Due to substantial scan-to-scan (or pixel-to-pixel) possible variation of a number of ions (charges) in the ICR cell, peak shifts may be prominent (space charge influence). Therefore, internal re-calibration is typically required for MSI datasets acquired with any FTMS instrument as calibration parameters vary from pixel to pixel. Performing a single point (lock mass) re-calibration is the simplest method employed. More sophisticated methods include several known peaks, as for example implemented for MALDI FT-ICR MSI by Smith and co-workers^41^. Another approach employed a number of ions present in the ambient laboratory environment, namely polydimethylcyclosiloxanes, as suggested by Barry and co-workers^42^. A number of mass spectra re-calibration procedures are known in general for FTMS non-imaging applications, as published and reviewed elsewhere^43,44^. In this work, we adapted one of the recent algorithms for re-calibration of FTMS mass spectra that is based on an iterative use of binomial averaging for calculating a non-linear re-calibration function^45^.

By applying all of the above to lipid imaging of both brain and kidney tissue on a hybrid 7 Tesla LTQ FT-ICR MS instrument we demonstrate the power of ultra-high mass resolution MSI for biological tissue imaging.

## Results

### Influence of ion accumulation time on mass accuracy and signal-to-noise ratio

An ICR cell requires a pre-set number of charges (typically 10^5^–10^6^) to be acquired for each scan (pixel) to achieve maximum analytical performance, both in mass accuracy and sensitivity^46^. To a point, larger ion populations in the ICR cell lead to higher sensitivity. Routine mass calibration is therefore performed using continuous electrospray infusion with automatic gain control (AGC) set to accumulate a fixed number of charges in the ICR cell (5 × 10^5^ in our case). In contrast, DESI MSI experiments are typically performed in the absence of AGC to ensure equidistant pixel distribution in an image and that the number of ions collected are representative of the given sampling region. Additionally, an accurate estimation of a number of charges for application of the AGC function is jeopardized because heterogeneous tissues inevitably produce fluctuating ion yields, which results in over- or under-filling of the ICR cell throughout an MSI experiment. This leads to characteristic *m/z* shifts, due to space charge effects^47^. If the average pixel-to-pixel *m/*z shift is greater than the *m/z* difference between isobaric compounds of interest, it is not possible to generate clear mass spectral images of these compounds. It is therefore of importance to ensure that, for most pixels, the ICR cell is filled within the optimal range of charge numbers.

**Figure 1:**
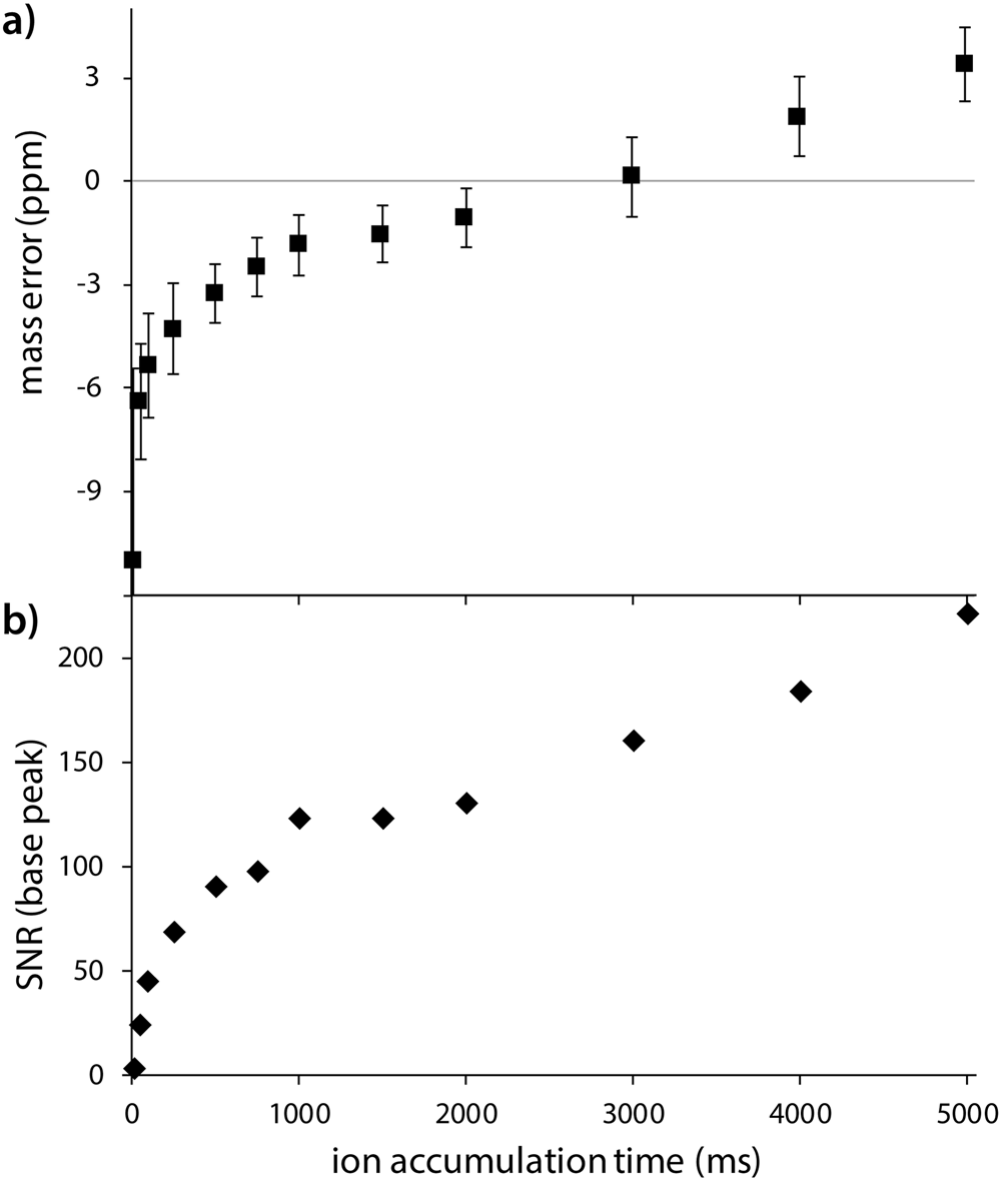
Method development on a DESI 7 T LTQ FT mass spectrometer. (**a**) Plot showing the measured 4-peak average mass error and (**b**) the base peak signal-to-noise ratio (SNR) with varying ion accumulation time. Data collected via subsequent line scans across a rat brain tissue section. Values shown are calculated from the averaged mass spectrum via Thermo Xcalibur Qual Browser with SNR calculated on the base peak and mass error calculated as the average error for four highly abundant lipid species (details in Supplementary Table S1).

Therefore, we first investigated the influence of ion accumulation time on both signal-to-noise ratio (SNR) and on parts-per-million (ppm) mass error relative to the default calibration optimised for 5 × 10^5^ charges target on a hybrid 7 Tesla LTQ FT-ICR MS instrument (Fig. 1). Averaged DESI mass spectra were recorded from consecutive rows on a (relatively) homogenous area of a rat brain section. The length of each row and the number of scans was kept the same (8.6 mm, 65 scans), while the stage speed was adjusted inversely with the ion accumulation time to ensure the pixel size was the same for each row. The base peak SNR and the mass error for four abundant lipid species (Supplementary Table S1) were calculated using the full averaged mass spectrum of each experiment.

As can be seen in Fig. 1a, larger ion numbers result in a mass shift towards higher *m/z* values for a constant calibration function, whereas negative mass errors are observed at low accumulation times. The smallest mass error was found at an ion accumulation time of 3000 ms. Based on this data and the fact that the instrument is calibrated for 5e5 charges we can estimate an ion collection rate of ~1.6 × 10^5^ ions/second in our experiment, assuming a constant generation rate and singly charged ions. It was observed that the SNR increases with injection time (Fig. 1b), flattening off towards longer accumulation times. This was expected, as DESI is able to generate stable signal from a single tissue location for at least several seconds. Most importantly, the optimal ion accumulation time range for maximum sensitivity under these experimental conditions turned out to be from 1000 till 4000 ms, much longer than typically used. Of course, larger ion populations increase the chance of peak coalescence between isobaric species with minimal mass difference. To separate minimal mass differences sensitivity might need to be sacrificed, as will be demonstrated below.

### Increased Duty Cycle and Mass Resolution per Unit Time via Modified Acquisition Sequence

The commercial LTQ-FT system does not support accumulation of ions in the external ion trap during acquisition of the transient signal in the ICR cell, as discussed above. In regular electrospray experiments, with ion accumulation times of <100 ms and typical transients in the order of hundreds of milliseconds, this does not significantly prolong analysis time. However, in ambient FT-ICR imaging such as DESI-MSI, both the optimal ion accumulation times and the acquisition times can be in the order of seconds, as shown above. In this case, parallel ion accumulation and signal acquisition provides immediate gains in throughput or both in sensitivity and mass resolution.

**Figure 2:**
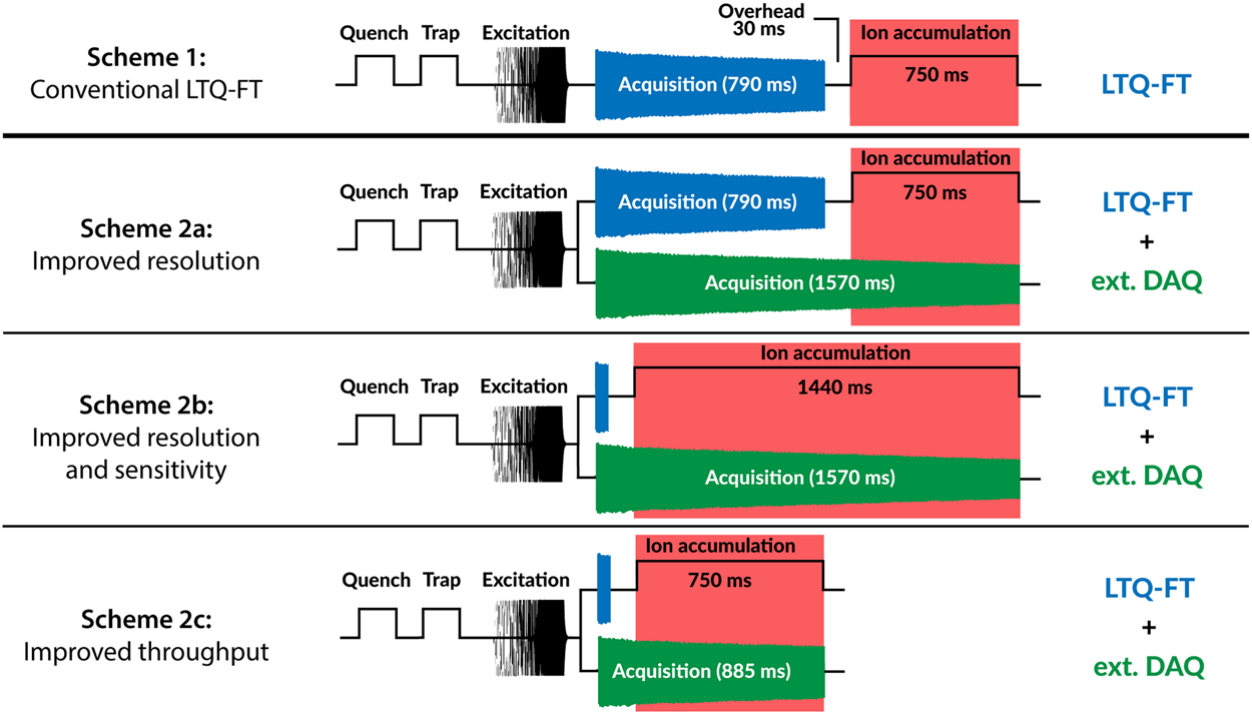
Accumulation and transient acquisition scheme options. (1) conventional acquisition at a set mass resolution of 100 k at *m/z* 400; (2**a**) parallel acquisition via the conventional scheme and external data acquisition (DAQ) unit FTMS Booster X1, the latter providing an estimated 220 k resolution in magnitude mode and 430 k in absorption mode at *m/z* 400; (2b) parallel acquisition via the external DAQ unit, optimized for both mass resolution and sensitivity; (2c) parallel acquisition via the external DAQ unit, optimized for throughput without sacrificing sensitivity or mass resolution compared to Scheme 1. *The timespans indicated for acquisition and accumulation are typical examples at 100 k mass resolution instrument setting (at *m/z* 400).

Figure 2, Scheme 1 shows a single acquisition cycle of the original LTQ-FT mass spectrometer. Typically, after the ICR cell quench, followed by a next ion population trapping and excitation events, the transient is recorded for a set period (correlated to the desired resolution). Once the ion detection is over, and after some overhead time required for electronic and on-the-fly data processing, the accumulation of ions for the next pixel is started and the sequence is repeated. In our approach, recording of the transient is performed in parallel with the standard LTQ-FT data acquisition by using an external high-performance data acquisition (DAQ) unit (Fig. 2, Schemes 2a–c). By doing so, the external transient acquisition continues whilst ions are accumulated for the next pixel; the transient recording continues up until the ICR cell is quenched to prepare for the next analysis cycle. This results in a longer transient (and thus a higher mass resolution) for the same overall scan time. Due to the advanced electronics architecture in the external DAQ system, the length of the transient can match any actual total ion detection time. That favourably compares to the previous generation DAQ systems, as discussed in Introduction.

Figure 2, Scheme 1 displays a conventional DESI-LTQ-FT analysis workflow (750 ms ion accumulation time, 100 k mass res. in 1.6 second scan time per pixel). With these settings, adding an external DAQ approach provides an estimated 2-fold gain in mass resolution with improved sensitivity at identical throughput by doubling the transient length (Fig. 2, Scheme 2a). However, the external DAQ unit can be used much more efficiently: the ion accumulation time can be lengthened to approximately 90% of the total scan time without adding to the total scan time by reducing the instrument resolution setting to 12.5 k, as shown in Fig. 2, Scheme 2b. Extending the ion accumulation time in this way improves the sensitivity by about 25% (Fig. 1) without compromising in mass resolution or throughput. Alternatively, the parallel operation can be used to increase the analysis throughput without sacrificing sensitivity or mass resolution, as shown in Fig. 2, Scheme 2c. In this way, the resulting sensitivity is identical to that obtained via Scheme 1, while the theoretical mass resolution is more than doubled (~230 k at *m/z* 400) even though the analysis time is halved.

### High-throughput, high-mass resolution imaging

**Figure 3:**
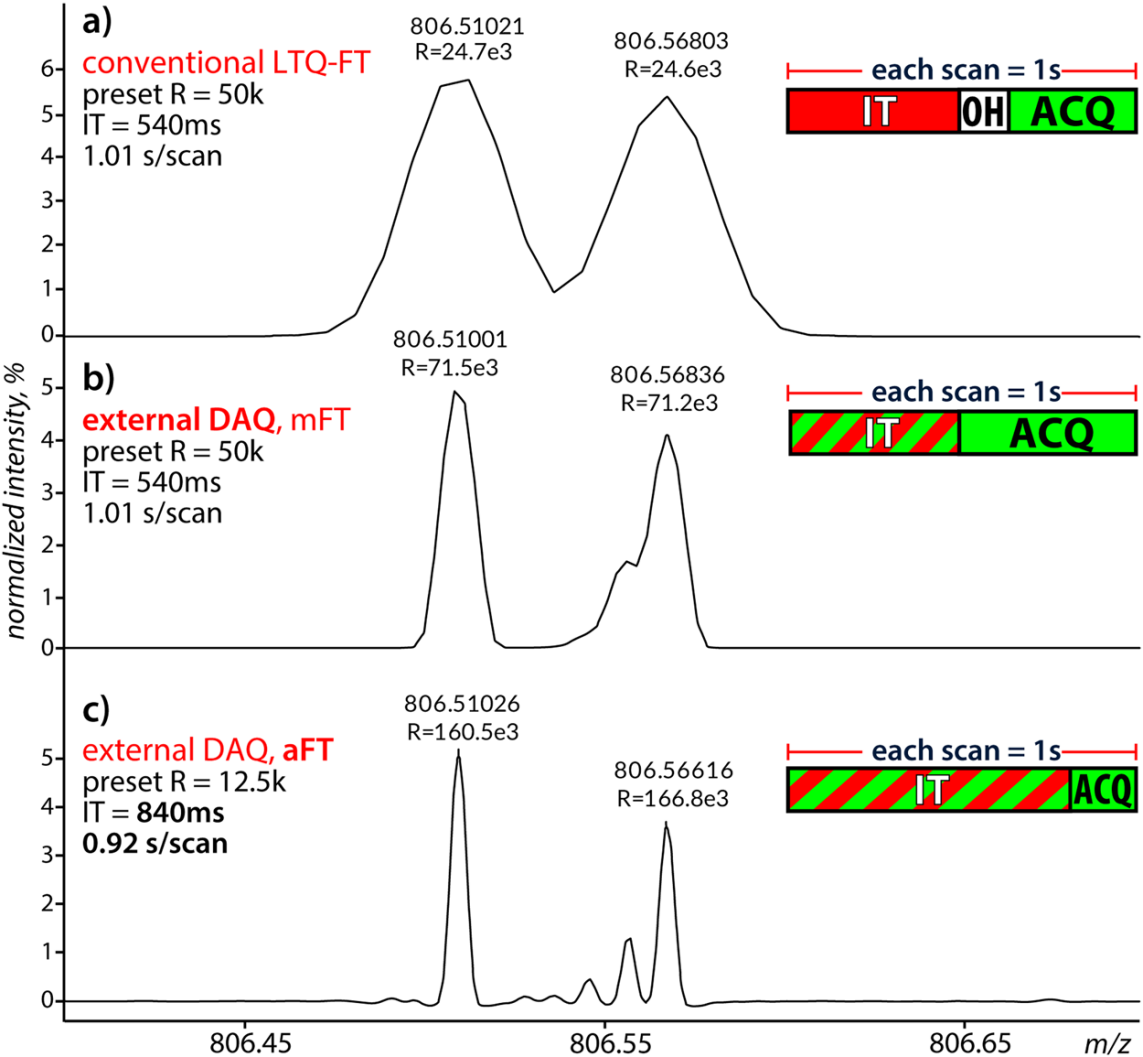
Mass spectral quality comparison between recording and processing modes for 1 pixel per second analyses of a rat kidney section. Zoom-ins of full image average reduced profile spectra of: (**a**) typical LTQ-FT performance at 50 k (at *m/z* 400) resolution setting; (**b**) in parallel acquired spectrum on the external data acquisition (DAQ) unit and obtained with mFT processing; (**c**) resolution and sensitivity optimized experiment for the external DAQ unit, with aFT processing. *IT = accumulation time; OH = overhead time; ACQ = acquisition time. Spectra are normalized to the spectrum base peak. The full profile aFT version of (**c**) is provided as Supplementary Fig. S1.

The analytical benefit of our approach is demonstrated in Fig. 3, showing spectra generated from rat kidney tissue using three different acquisition sequences. Figure 3a corresponds to the conventional workflow as shown in Fig. 2, Scheme 1. The conventional LTQ-FT was optimized for a “fast” imaging experiment at 1 scan per second. As expected, the measured mass resolution was ~25 k at *m/z* 800, in accordance with the 50 k at *m/z* 400 specifications. Simultaneously, the dataset displayed in Fig. 3b was acquired on the external DAQ unit and FT processed in magnitude mode (mFT). The resulting 71 k (at *m/z* 800) mass resolution shows a significant (2.9-fold) improvement over the spectrum recorded using the conventional system for an identical scan time. The increase in resolving power reveals some additional mass spectral features. When this experiment is repeated on an adjacent tissue section, but now with sensitivity and resolution optimized similar to Fig. 2, Scheme 2b, and absorption mode FT processing (aFT) is used instead of mFT processing, all features are baseline separated with a mass resolution of 160 k (Fig. 3c). A 6-fold overall improvement in mass resolving power compared to the conventional approach is the result without sacrificing throughput.

**Figure 4:**
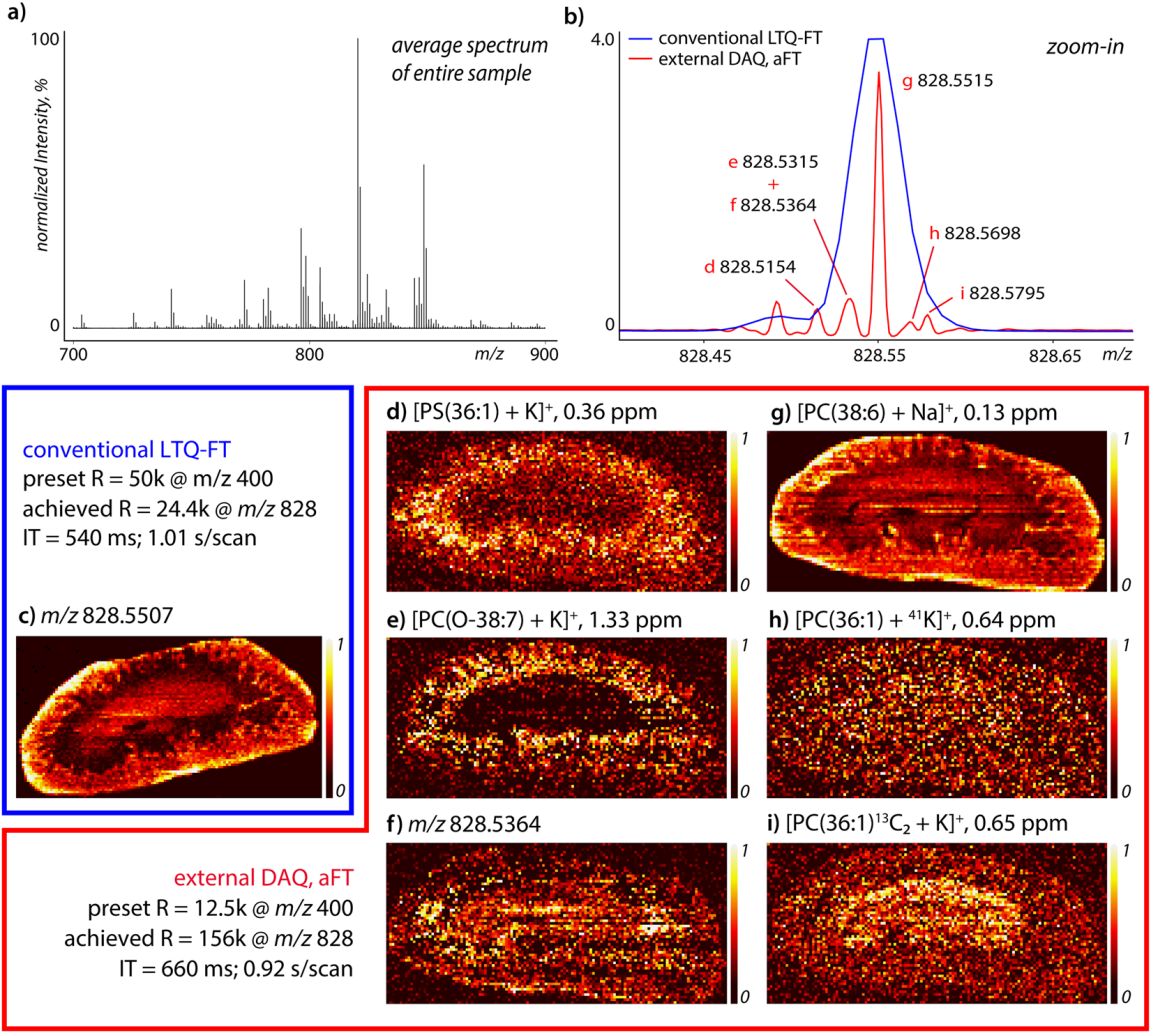
Mass spectral images of lipid species detected around *m/z* 828.55 in ~1 scan per second experiments on adjacent rat kidney sections. Top-right shows the spectrum around *m/z* 828.55, for the conventional LTQ-FT setup (blue) and the external DAQ (red). The achieved mass resolution on the most abundant *m/z* 828.55 peak was 24.4 k and 156 k, respectively (average spectrum of entire sample area). Image recording took 2 hours for each image at 150 µm by 150 µm pixel size, with both datasets containing approximately 8000 pixels. All images are TIC normalized and plotted on a linear intensity scale. Given the very low abundance of some species, a 5% image intensity threshold was applied to each image to reduce noise. The full profile aFT version of (**b**) is provided as Supplementary Fig. S2.

To demonstrate the value of our approach for MSI, we show examples of ion distribution images from the first (Fig. 3a) and third (Fig. 3c) dataset in Fig. 4 (note these are low abundance ions with relative intensities 0.5–4%). The average spectrum is shown in Fig. 4a. The single peak detected at *m/z* 828.55 in the conventional LTQ-FT setup (Fig. 4b, blue) revealed six isobaric species in the external DAQ approach, with aFT processing (Fig. 4b, red). Of these six species three could be tentatively identified as monoisotopic lipid species and two as isotope peaks (^41^K and 2x^13^C) of phosphocholine 36:1 (PC(36:1)). Species *m/z* 828.5364 (Fig. 4f) was not fully resolved from *m/z* 828.5315 (Fig. 4e) but each reveals a clearly distinct spatial distribution. Both datasets were uploaded to METASPACE^48^ and processed against the HMDB-v4 database with a maximum FDR of 10%, a minimum metabolite-signal match (MSM) score of 0.5 and a mass error ≤3 ppm^49^. The conventional LTQ-FT dataset resulted in 34 annotations in the 700–900 *m/z* range versus 66 annotations for the external DAQ aFT dataset.

### Exploring limits of high mass resolution DESI imaging

As can be seen in Fig. 4, the use of high mass resolution in DESI imaging unravels a range of nearly isobaric (cat)ionization variants and overlapping isotope species. Minute mass differences, such as the difference between ^13^C and CH (4.5 mDa) or SH_4_ and C_3_ (3.4 mDa), can be resolved by taking advantage of the high mass resolving power offered by FT-ICR MS. The combined resolving power and mass accuracy supports confident assignment of elemental compositions of biological species. And by resolving more spectral features, the spatial distribution of an increasing variety of distinct chemical species can be revealed, as demonstrated in Fig. 4.

One of the challenges left to resolve is the distribution of different cationized lipid adduct species. For example, changes in ratios of adduct species (e.g., sodiated and potassiated adducts of PC lipids) can infer insight into altered biochemical processes in tissues. As an example, a reduction in PC [M + K]^+^ ions was correlated with an increase in [M + Na]^+^ at the site of traumatic brain injury in mice and attributed to loss of Na^+^/K^+^-ATPase activity^50^. Unambiguous detection of individual adduct species can be challenging as the accumulated mass defects are usually compounded by the presence of different monoisotopic or isotope peaks. For this reason, we have explored the current limits in high mass resolution DESI imaging by resolving the *m/z* difference between the sodiated species of phosphocholine (34:1) [PC(34:1) + Na]^+^ and the protonated species of phosphocholine (36:4) [PC(36:4 + H]^+^. Here, both species are separated only by 2.4 mDa, or 3.1 ppm. To properly resolve this difference a mass resolving power of approx. 900 k at *m/z* 782 is theoretically required, assuming equal peak height, as shown in Supplementary Fig. S3, generated using a method described earlier^51^. Here, properly resolved is defined as achieving the deepest possible valley between the two peaks (see Supplementary Video S1).

**Figure 5:**
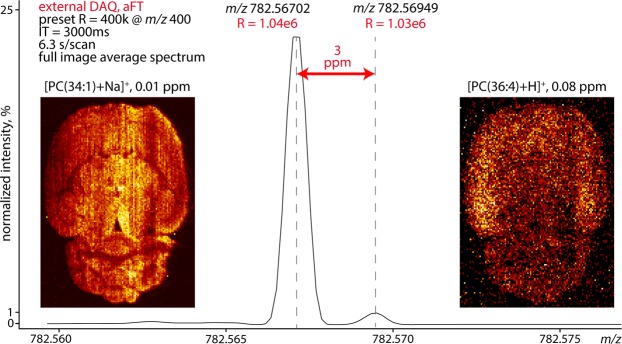
Full image average mass spectrum showing the separation of [PC(36:4 + H]^+^ and [PC(34:1) + Na]^+^. The data was acquired on the external DAQ unit and processed with aFT. Acquisition time of 16761 spectra at a pixel size of 150 µm was approximately 31 hours. Insets: the spatial distributions of [PC(34:1) + Na]^+^ and [PC(36:4 + H]^+^, noted with the annotation mass error. The images are TIC normalized and spectral abundance is indicated as relative to the base peak (*m/z* 798.5) in the full mass range (*m/z* 700–900). Note the achieved mass resolution (1 million@*m/z* 782).

We acquired an image dataset of a rat brain section with an acquisition time per pixel of ~6.3 seconds. Figure 5 shows the baseline separation of [PC(34:1) + Na]^+^ and [PC(36:4 + H]^+^ species with a mass resolution of ~1 M in the full image average spectrum (Supplementary Fig. S4). The insets of Fig. 5 show the spatial distribution of PC(34:1) and PC(36:4) across the rat brain section. PC(34:1) is quite homogenously distributed, whereas PC(36:4) shows higher abundance near the cortex but is nearly absent in the cerebellum. To generate clear MS images of these two species, care had to be taken to avoid any space-charge and peak coalescence effects. Therefore, the ion population in the ICR cell was intentionally kept low by restricting the ion accumulation time to 3000 ms, restricting the mass range to *m/z* 765–832 and reducing the solvent flow rate to 3 µl/min (in contrast to 5 µL/min used for datasets shown above). Naturally, reducing the ion populations in the cell lowered the dynamic range of the experiment to about two orders of magnitude, as shown by Fig. 5. Total acquisition time for this dataset was 31 hours, however future adoption of this approach to higher magnetic field and/or frequency multiple detection would reduce the acquisition time (see Introduction). A comparison of data file sizes is included as Supplementary Table S3. Measured mass accuracy (after internal recalibration) of these two ions throughout the experiment is shown in Supplementary Fig. S5. Analogous separation and distinct ion distribution imaging of sodiated phosphocholine (36:1) and protonated phosphocholine (38:4) are provided in Supplementary Fig. S6. The corresponding mass errors are provided in in Supplementary Fig. S7. Example ion distributions of additional lipid species are provided in Supplementary Fig. S8.

## Discussion

We have demonstrated an approach to drastically improve the sensitivity, mass resolution and/or throughput of DESI-FT-ICR MS imaging experiments on a 7 Tesla LTQ-FT instrument. For the first time, absorption mode FT processing has been applied in an ambient imaging setting and its benefits (about 2-fold resolution increase over magnitude mode) have been shown. We have shown that our parallel data acquisition approach makes it possible to achieve 6-times higher mass resolution in the same experimental time period, which is crucial for imaging approaches. In 1 scan/second experiments isotopic lipid patterns could be revealed (Fig. 4), demonstrating that in positive mode DESI-imaging at conventional mass resolution the spatial distributions of multiple isobaric lipid species merge, which will almost inevitably lead to incorrect interpretation. Enabling ion accumulation for extended periods of time benefits specifically the sensitivity for continuous ionization methods, such as DESI MSI. In the case of MALDI MSI where the time needed to fully deplete a given sampling position can be 10–100-fold shorter (10–100 s of milliseconds), the benefit of performing parallel ion accumulation and detection is reduced. Nevertheless, available extended periods of ion accumulation can be beneficial also for MALDI and other pulsed ionization methods when increased sensitivity is required, including for MS/MS applications where precursor ions from multiple adjacent sampling regions may be accumulated prior to fragmentation for enhanced sensitivity. Furthermore, it shall be noted that the presented here approach enables ion detection also in parallel to other processes external to the ICR cell, not only to ion accumulation. That includes, among others, an overhead time that is required for on-the-fly data processing, which may take >100 ms and more per pixel in a conventional MALDI FT-ICR MSI experiment.

MSI with one million mass resolution was demonstrated for a variety of lipid species, which resolved some of the smallest mass differences encountered in lipid imaging of biological tissues. To the best of our knowledge, this is the highest mass resolution imaging dataset reported to-date. We have shown that ultra-high mass resolution can increase the information content of imaging experiments and can add confidence to the validity of experimental results. It must be noted that all annotations provided in this manuscript are limited to lipid class, total chain length and double bond count (i.e., sum-composition level), which is the limit of high mass resolution and accuracy in the absence of MS/MS data^52^. However, by using ultra-high resolution imaging confidence in mass spectral annotation is greatly increased and many more “pure” sum-composition level images are obtained, as demonstrated by the METASPACE annotation queries. Moreover, with the recent demonstration of intact protein imaging with DESI^53^ our approach could readily be applied to MSI spectral performance for protein imaging, where increased sensitivity and identification of charge states of large, highly charged protein ions are key steps towards improved protein identification.

To enable high mass resolution ambient imaging the accumulation of ions, acquisition of FT-ICR MS transients, and allied data processing approaches need to be optimal. Our approach extracts exceptional performance from a standard 7 Tesla LTQ-FT instrument equipped (without hardware and data acquisition software modifications) with an external DAQ system, delivering unprecedented sensitivity, mass resolution and speed. This approach is applicable to other types of FTMS instruments, including other designs of FT-ICR MS and Orbitrap FTMS instruments, where it may provide comparable advantages for all or for certain parts of the imaging workflow presented here. The authors feel that the ability to record and store the transient in high quality and full length is crucial, as it will enable even higher performance as data processing methods become more sophisticated. Currently, endeavours to push (ultra-) high mass resolution imaging are still scarce, for mostly practical reasons. Until now, there were no commercial or open-source solutions available capable of working with MSI data of this quality. Specifically, the developed imaging software allowed processing image datasets of any size, with an example of an up to 1 TB image processed in the current work, without image splitting into a number of smaller chunks or using a mass resolution reducing “binning” method. We feel confident this work will help to make ultra-high mass resolution imaging more accessible to the field.

## Methods

### Materials

Sections of 12 µm thickness were cut from fresh frozen healthy rat brain (transverse) and kidney (coronal) using a cryo-microtome (HM525; MICROM Walldorf, Germany) at -19 °C and -21 °C, respectively. These sections were thaw-mounted onto plain glass slides and stored at -80 °C until further processing. The mounted sections were placed in a desiccator for 20 minutes to remove excess water prior to analysis. HPLC-grade methanol and formic acid 99% (Biosolve B.V., Valkenswaard, The Netherlands) were used for the DESI spray solvent.

### Desorption electrospray ionization (DESI) mass spectrometry

All MSI experiments were performed using a 2D-DESI source (Prosolia Inc., Indianapolis, IN, USA) coupled to a 7 Tesla LTQ-FT Classic mass spectrometer (Thermo Fisher Scientific, Bremen, Germany). That implies an original, prior to Ultra ICR cell design, ICR cell configuration was employed. All images were recorded with a 150 µm pixel size. To ensure square pixels were acquired, the continuous stage motion speed was set to cover 150 µm during each single scan. The ion accumulation time — automatic gain control (AGC) was disabled — was optimized prior to analysis, depending on the experimental requirements.

All data were recorded in positive ion mode with mass spectrometer settings as follows: tube lens voltage of +100 V, capillary voltage of +44 V, and a capillary temperature of 320 °C. DESI was performed with an electrospray voltage of +5 kV and 7.0 bar nitrogen nebulizing gas pressure. The spray solvent consisted of methanol with 0.7% formic acid and was delivered via syringe pump at a flow rate of 5 µl/min (with the exception of 3 µL/min to generate the data shown in Fig. 5 and Supplementary Figs 4–8).

### Parallel data acquisition

The instrument was coupled to an external high-performance data acquisition (DAQ) system (FTMS Booster X1, Spectroswiss, Lausanne, Switzerland) to record the transients in parallel with the LTQ-FT signal processing unit^31^. The employed DAQ system was developed on the platforms of high-performance field-programmable gate array (FPGA) and PXI-Express technologies. The system combines, on a high data transfer speed chassis, a high sampling frequency digitizer with an FPGA chip onboard for high-throughput in-line digital signal processing (DSP) algorithms, a dedicated computer for data co-processing, and an amplifier for signal conditioning. The DSP algorithms on the FPGA chip include a low-jitter digital decoder that detects start and stop triggers from the host instruments, enabling full transient acquisition. The DSP algorithms were developed and deployed to the FPGA using Xilinx compilation tools and LabVIEW (National Instruments, Ennetbaden, Switzerland). The DAQ system is interfaced to an FTMS instrument of interest via standard FTMS digital and analog output connectors. Recorded data files are integrated with metadata from the host FTMS through software interfaces to vendors’ file formats, e.g.,.RAW. The external DAQ unit records transient in parallel to the built-in electronics, which enables evaluation and comparison between datasets. Recording transients at higher quality allows for more sophisticated processing, resulting in higher quality mass spectra compared to the original system. The source, instrument and external DAQ unit were synchronized and controlled via Omnispray 2.0.1 (Prosolia Inc.), Xcalibur 2.1 (Thermo Fisher Scientific) and FTMS Booster Ctrl (Spectroswiss), respectively.

### Data processing

Mass spectra and images were generated from the recorded transients using Peak-by-Peak software for data processing and conversion. AutoVectis software was used for absorption mode Fourier transform (aFT) signal processing. A newly developed feature in the AutoVectis software enabled efficient (aFT) processing on whole imaging datasets acquired with LTQ-FT coupled with the FTMS Booster data acquisition systems for the first time^54^. Full apodization was used to generate absorption mode spectra with no long-range baseline deviation. The F-value for the absorption mode processing was equal to 0.5, with 3 zero pads (fills). Note that the conventional LTQ-FT data acquisition and analysis approach exclusively uses magnitude mode Fourier transform (mFT) processing, whereas the external DAQ unit and associated processing/analysis software enables the use of aFT processing for imaging. The conventional LTQ-FT data is acquired following the standard procedures and stored as reduced profile mass spectra in Thermo RAW format. Both datasets are then converted into HDF5 file format for further processing using Peak-by-Peak. Following additional developments performed for the current manuscript, both imaging data processing and analysis software packages, Peak-by-Peak and AutoVectis, are now available commercially (Spectroswiss).

Noise thresholding, internal recalibration, peak picking and image generation was performed using the newly developed Peak-by-Peak imaging software. Noise thresholding level was determined as standard deviation of noise multiplied by a user-defined factor. The standard deviation was calculated by using a data-dependent noise thresholding algorithm^55^.

Each pixel of a whole dataset was internally re-calibrated using a reference mass list (Supplementary Fig. S9). The selected reference masses are well known, highly abundant lipid species spread over the lipid mass range of interest (Supplementary Table S2). The non-linear mass re-calibration method is described in Kozhinov *et al*.^45^. Search of an experimental mass in a single mass spectrum was performed within a mass tolerance window with its centre at the corresponding reference mass. To exclude the picking of parasitic sidebands and side-lobes instead of analyte peaks, which can potentially disturb mass-recalibration, the highest peak in the mass tolerance window was selected. Single or several re-calibration iterations with different mass tolerance windows (±2–30 ppm) were performed depending on the quality of initial (external) mass calibration and influence of space charge effects on frequency deviation.

### Image generation

Image generation was performed by selecting the highest peak within a preselected mass tolerance window around the requested image mass value for each pixel. The mass tolerance window was adjusted to match the accuracy of the dataset. Parallel (multi-core) calculations, including 3-point interpolation peak picking of a reduced profile mass spectrum and a finding of peaks corresponding to the requested mass values in each pixel, were performed for the generation of images each time. This method allowed processing image datasets of any size, without loading all the data into the RAM memory, and was found to be more suitable for analysis of ultra-high mass resolution datasets than the prevalent “binning” method of MSI processing. Additionally, the distribution of mass errors between the requested and experimental mass values calculated in each pixel was plotted for an image, which helps to control image quality. The requested image mass value was adjusted in case the mean of mass error distribution was larger than 0.1 ppm. The resulting average image mass is shown in the spectra; its deviation from the annotated exact mass is the reported mass error value. All images shown are plotted on a linear intensity scale. All annotations given are tentative, based on measured *m/z* only, and are thus only reported to the sum-composition level.

## Supplementary information

Supplementary Information

Supplementary Video S1

## Data Availability

Two annotated datasets are made publicly available via METASPACE^49^. All datasets generated during and/or analysed during the current study are available from the corresponding author on request.
