# Supplementary material for: Increased throughput and ultra-high mass resolution in DESI FT-ICR MS imaging through new-generation external data acquisition system and advanced data processing approaches
Source: Sci Rep. 2019 Jan 9;9:8. doi: 10.1038/s41598-018-36957-1 (PMC6327097; doi:10.1038/s41598-018-36957-1)
Supplement: Supplementary file 1 — Supplementary Information [file 41598_2018_36957_MOESM1_ESM.pdf]

SUPPLEMENTARY INFORMATION

Increased throughput and ultra-high mass resolution in DESI FT-ICR MS imaging through new-generation external data acquisition system and advanced data processing approaches

Pieter C. Kooijman[1,2], Konstantin O. Nagornov[3], Anton N. Kozhinov[3], David P.A. Kilgour[4], Yury O. Tsybin[3], Ron M.A. Heeren[1] and Shane R. Ellis[1]*

[1]Maastricht University, Maastricht Multimodal Molecular Imaging Institute (M4I), Maastricht, 6229ER, The Netherlands
[2]TI-COAST, Amsterdam, 1098 XH, The Netherlands
[3]Spectroswiss, EPFL Innovation Park, 1015 Lausanne, Switzerland
[4]Nottingham Trent University, Department of Chemistry and Forensics, Nottingham, NG11 8NS, United Kingdom

Table S1: Lipid species used for determination of mass error in main text Figure 1a

ID	species	mol. formula	theoretical *m/z*
PC(32:0)	$[M+K]^+$	C40H80NO8P	772.5253
PC(34:1)	$[M+Na]^+$	C42H82NO8P	782.5670
PC(34:1)	$[M+K]^+$	C42H82NO8P	798.5410
PC(36:1)	$[M+K]^+$	C44H86NO8P	826.5723

Table S2: Exact mass values used for pixel-by-pixel internal calibration

ID	species	mol. formula	*m/z*
PC(34:1)	$[M+H]^+$	C42H82NO8P	760.585082
PC(36:0)	$[M+K]^+$	C40H80NO8P	772.525313
PC(34:1)	$[M+K]^+$	C42H82NO8P	798.540964
PC(36:4)	$[M+K]^+$	C44H80NO8P	820.525313
PC(36:1)	$[M+K]^+$	C44H86NO8P	826.572263
PC(38:6)	$[M+K]^+$	C46H80NO8P	844.525313
PC(38:4)	$[M+K]^+$	C46H84NO8P	848.556613
PC(40:6)	$[M+K]^+$	C48H84NO8P	872.556613

Table S3: File size comparison between vendor original reduced profile data and external DAQ data

Data type	File size (MB)
LTQ-FT reduced profile (.raw)	275
ext. DAQ aFT reduced profile (.h5)	3838
ext. DAQ transients (.h5)	763904

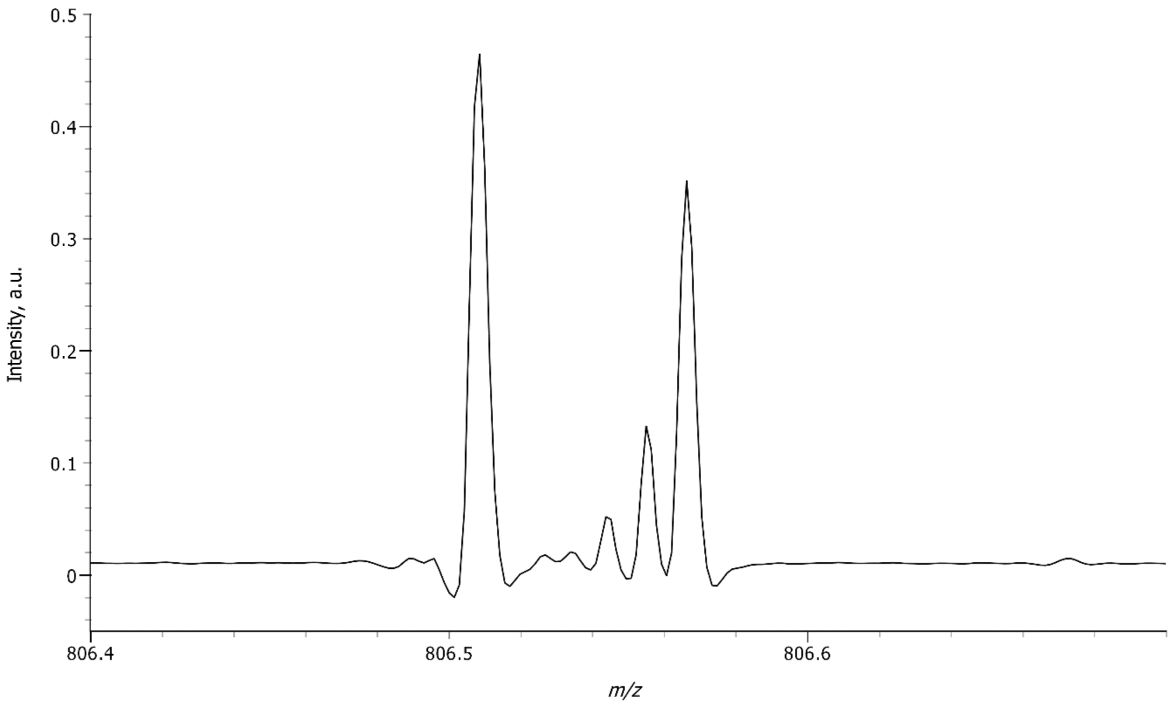

Figure S1: Full image average full profile aFT spectrum of Figure 3c in the main text.

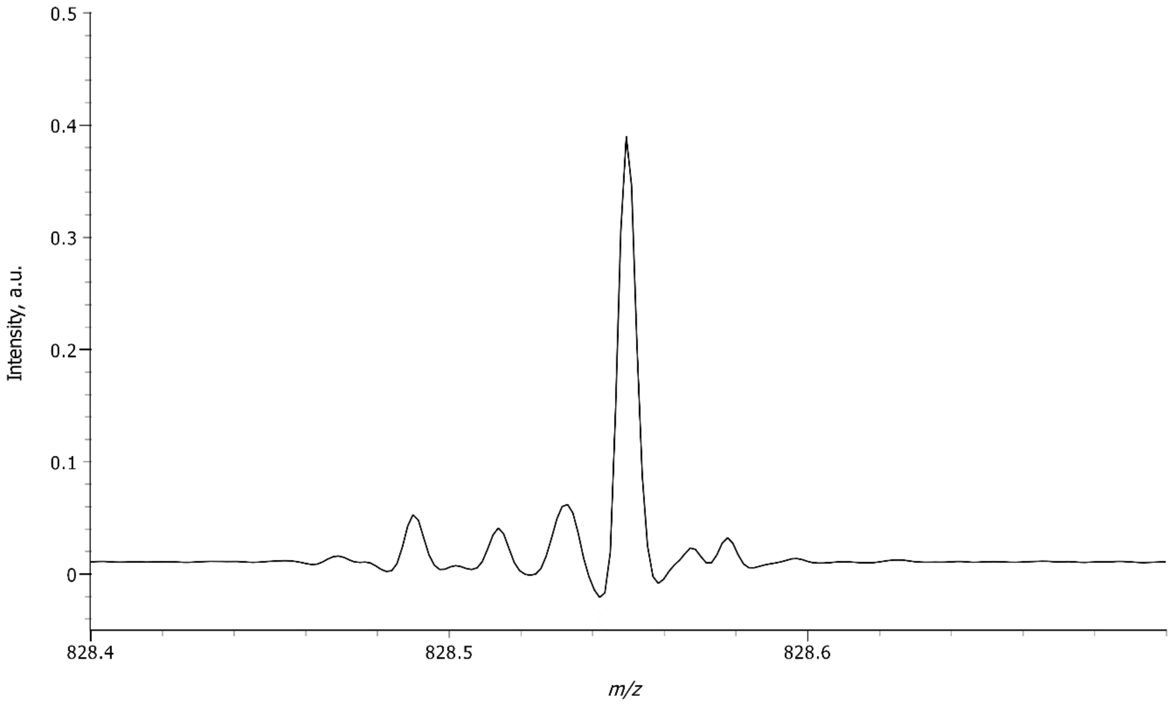

Figure S2: Full image average full profile aFT spectrum of Figure 4b in the main text.

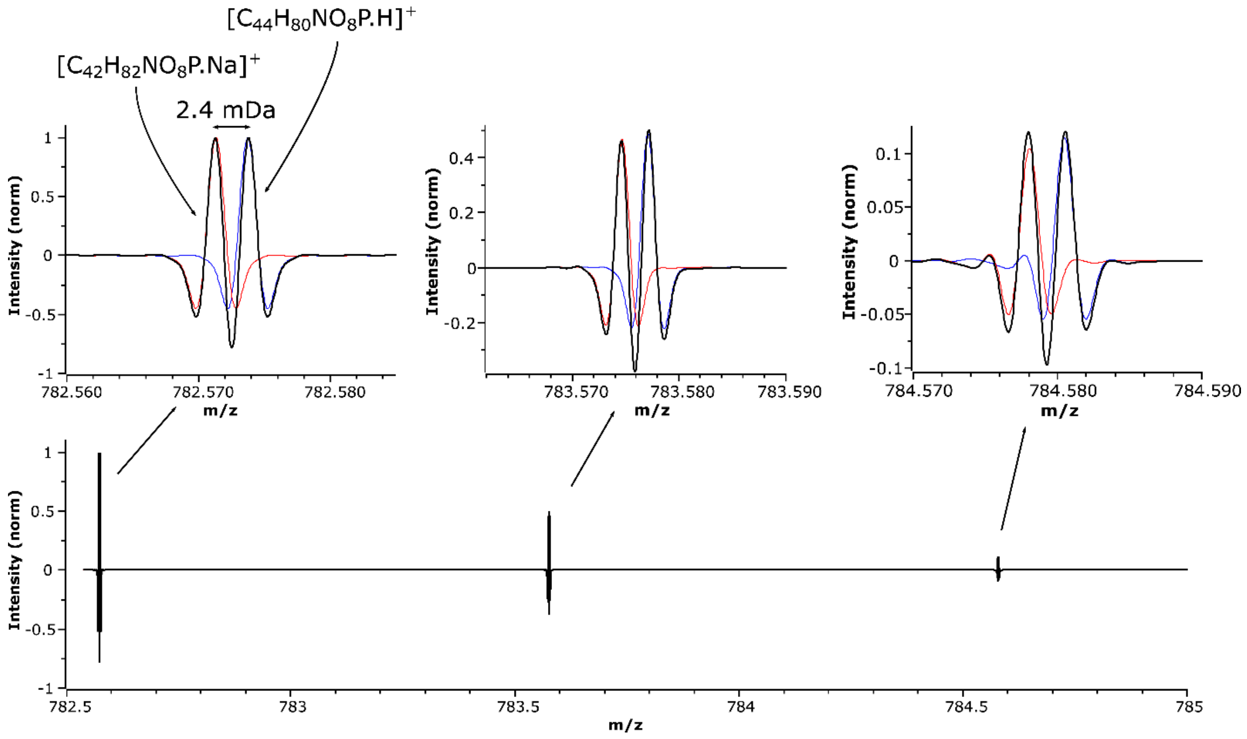

Figure S3: Simulated mass spectra of the sodiated species of phosphocholine (34:1) $[PC(34{:}1)+Na]^+$ and the protonated species of phosphocholine (36:4) $[PC(36{:}4+H]^+$ at a mass resolving power of 900k, assuming equal peak height.

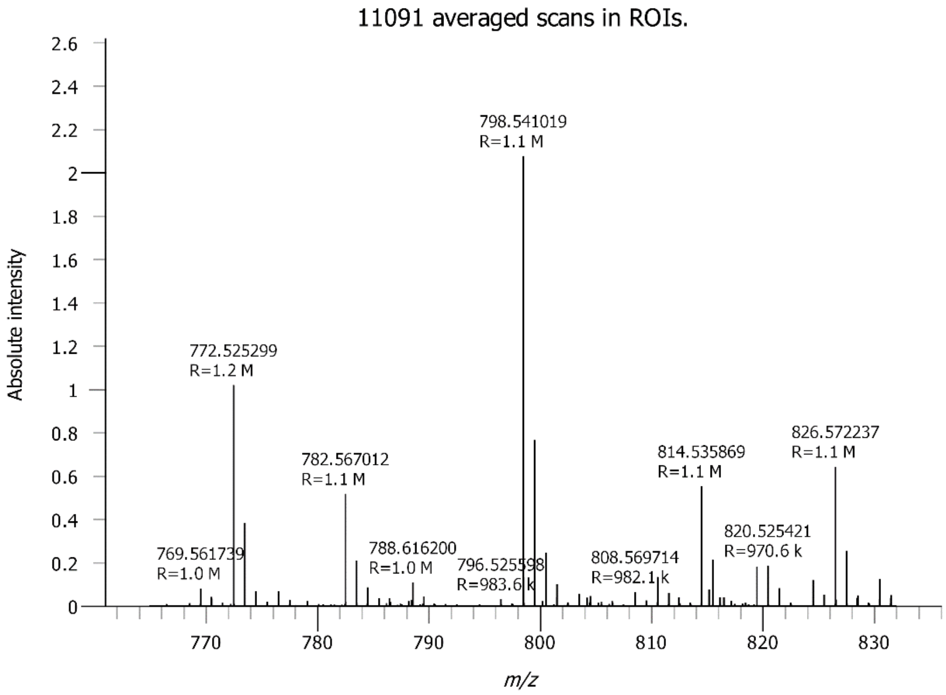

Figure S4: Full average mass spectrum of the rat brain imaging dataset presented in Figure 5.

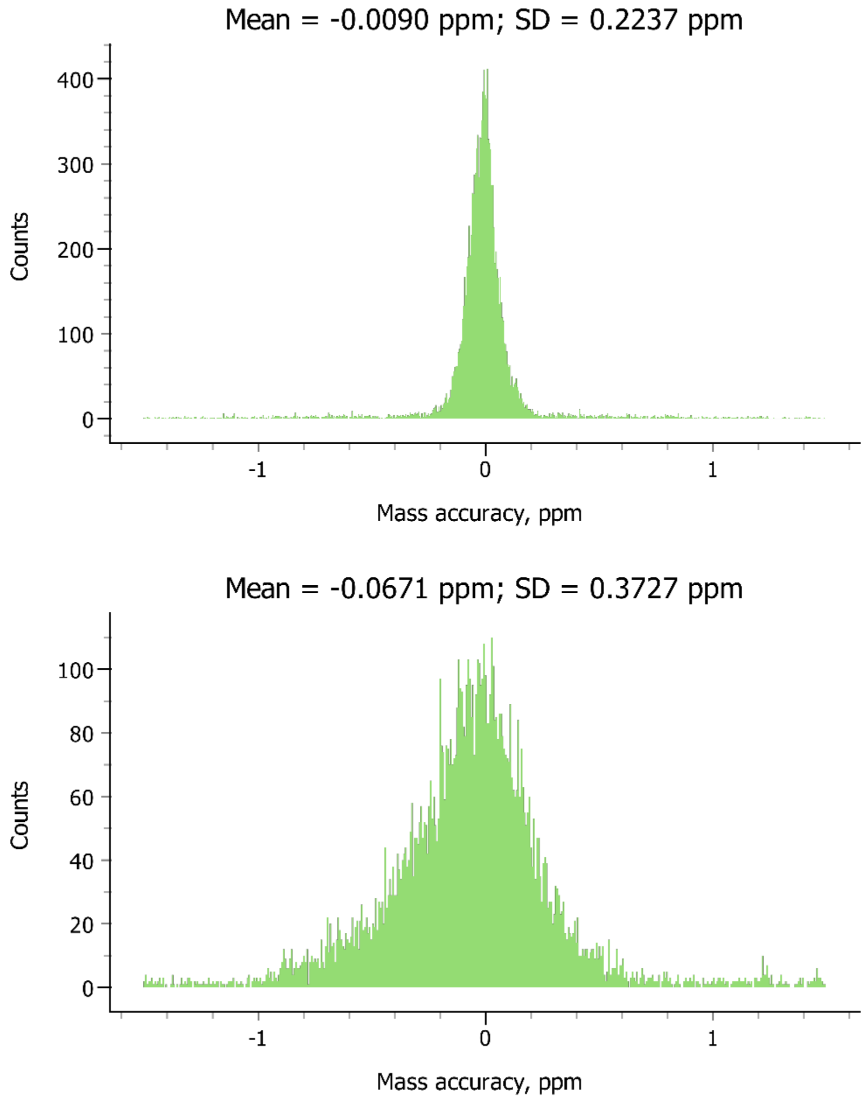

Figure S5: Mass error distributions for the image of (top) $[PC(34:1)+Na]^+$ and (bottom) $[PC(36:4+H]^+$. The corresponding images are shown on Figure 5 in the main text.

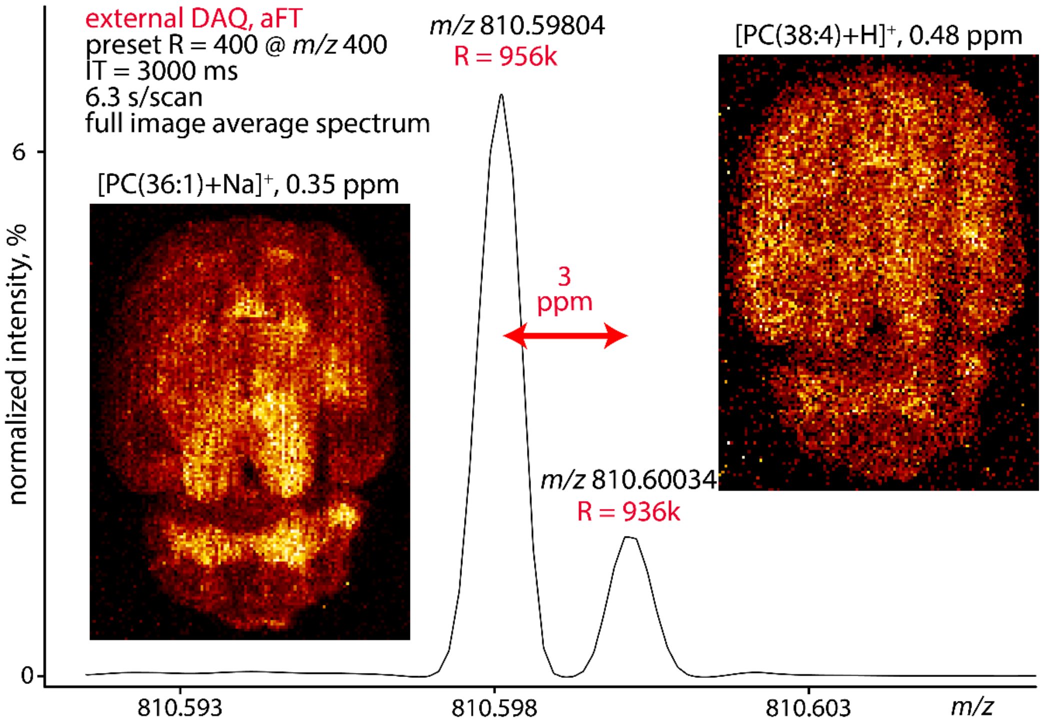

Figure S6: MSI separation of sodiated phosphocholine (36:1) from protonated phosphocholine (38:4). A narrow mass window of the full image average spectrum is shown.

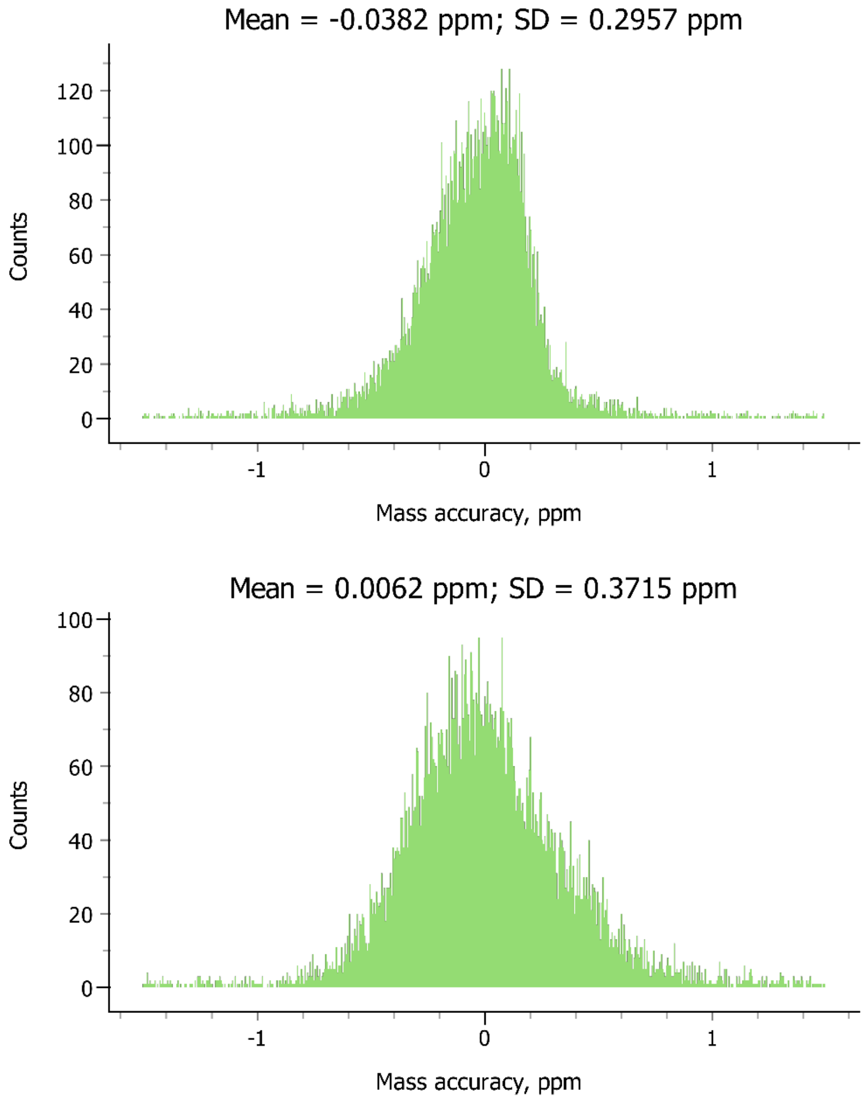

Figure S7: Mass error distributions for the image of (top) $[PC(36:1)+Na]^+$ and (bottom) $[PC(38:4+H]^+$. The corresponding images are shown on Supplementary Figure S4 above.

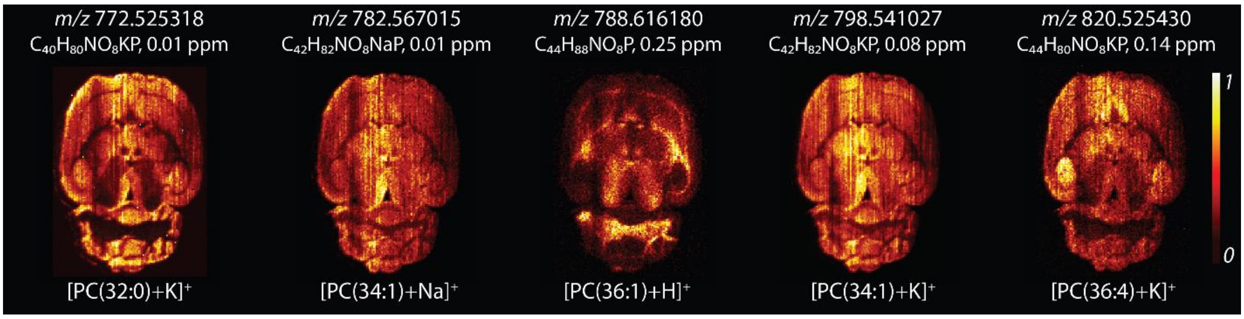

Figure S8: Relative distributions of five commonly observed lipid species obtained by DESI-FT-ICR MSI on a rat brain section. Images are plotted without normalisation and are from the same dataset used to prepare Figure 5 and Figure S6.

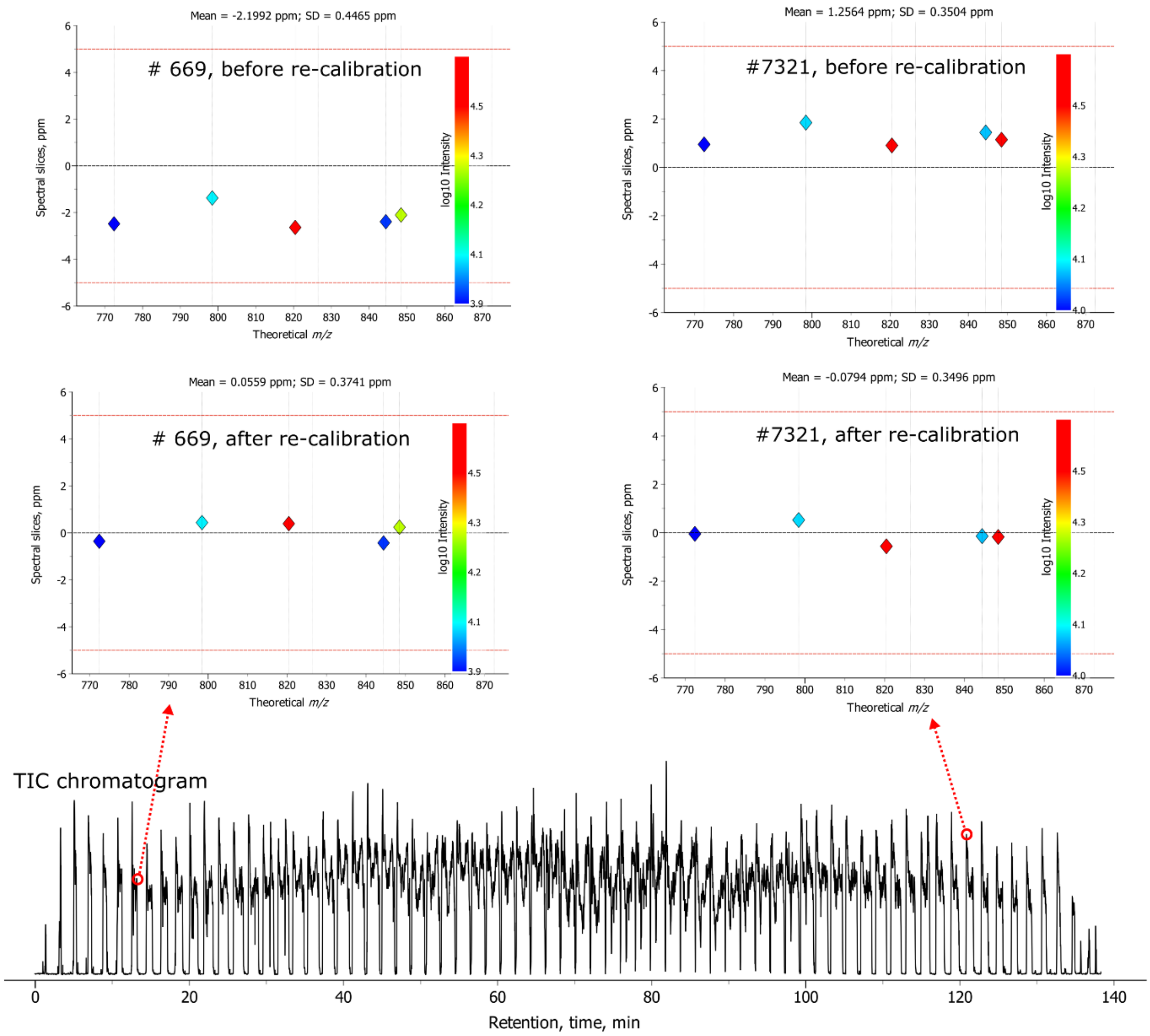

Figure S9: Pixel-by-pixel internal mass re-calibration of DESI imaging experiment of kidney tissue using the reference mass list in Table S2. (Bottom panel) total ion current (TIC) chromatogram of the experiment. Mass error distribution of found experimental masses corresponding to the reference ones in certain scan numbers (top panel) before and (middle panel) after mass re-calibration procedure.
